# Understanding variants of unknown significance and classification of genomic alterations

**DOI:** 10.1093/oncolo/oyae149

**Published:** 2024-07-09

**Authors:** Dean C Pavlick, Garrett M Frampton, Jeffrey R Ross

**Affiliations:** Department of Computational Discovery, Foundation Medicine, Inc., Boston, MA, United States; Department of Computational Discovery, Foundation Medicine, Inc., Boston, MA, United States; Department of Pathology, Foundation Medicine, Inc., Boston, MA, United States, and; Departments of Pathology, Medicine (Oncology), and Urology, Upstate Medical University, Syracuse, NY, United States

**Keywords:** pathogenicity, genomic variant, variants of unknown significance

## Abstract

Despite recent efforts to issue clinical guidelines outlining strategies to define the pathogenicity of genomic variants, there is currently no standardized framework for which to make these assertions. This review does not present a step-by-step methodology, but rather takes a holistic approach to discuss many aspects which should be taken into consideration when determining variant pathogenicity. Categorization should be curated to reflect relevant findings within the scope of the specific medical context. Functional characterization should evaluate all available information, including results from literature reviews, different classes of genomic data repositories, and applicable computational predictive algorithms. This article further proposes a multidimensional view to infer pathogenic status from many genomic measurements across multiple axes. Notably, tumor suppressors and oncogenes exhibit fundamentally different biology which helps refine the importance of effects on splicing, mutation interactions, copy number thresholds, rearrangement annotations, germline status, and genome-wide signatures. Understanding these relevant datapoints with thoughtful perspective could aid in the reclassification of variants of unknown significance (VUS), which are ambiguously understood and currently have uncertain clinical implications. Ongoing assessments of VUS examining these relevant biological axes could lead to more accurate classification of variant pathogenicity interpretation in diagnostic oncology.

## Teaser

This review does not present a step-by-step methodology but, rather, takes a holistic approach to discuss many aspects that should be taken into consideration when determining variant pathogenicity.

**Implications for practice**

The process by which pathogenicity is determined for genomic variants is convoluted and inconsistent. This report emphasizes different aspects of pathogenicity which should be considered for all variants in the context of oncology and highlights genomic measurements and rationale which should be incorporated into a final pathogenicity classification. Variants of unknown significance (VUS), which pose an inherent challenge in their clinical interpretation, can be examined using the proposed strategies.

## Introduction

Genetic testing to discover clinically relevant variants in a patient’s germline DNA and genomic testing to ascertain any relevant pathogenic driver and susceptibility mutations in tumor DNA have become routine in the clinical management of cancer patients. Precision oncology is marked by the identification of these patients who have historically lacked therapeutic opportunities, but with novel testing methodologies, have the potential to be assigned effective treatment with molecularly guided targeted antibodies, small molecules, or immunologic agents. HER2-targeted antibodies, such as trastuzumab, anti-ALK kinase inhibitors like lorlatinib, and anti-PD-1 checkpoint inhibitors like pembrolizumab, are just a few exemplars demonstrating significant survival benefits in each molecularly stratified cohort. This paradigm-shift toward both genomic characterization of an individual’s disease and targeted drug development is dependent on the success of diagnostic tools that can accurately identify molecular targets. However, as the adoption of multigene next-generation sequencing (NGS) diagnostic platforms continues to increase within the domains of genetic counseling, cancer detection, and targeted therapy selection, laboratories are presented with essential challenges that need to be addressed to ensure appropriate personalized patient care.^1^ This review focuses specifically on variant annotation in the setting of somatic testing in human cancers, though the importance of this in hereditary testing and non-cancer diseases is also critically important for patient management.

In 2015, the American College of Medical Genetics and Genomics (ACMG) and the Association for Molecular Pathology (AMP) issued guidelines outlining how detected variants are to be classified, either as pathogenic (P), likely pathogenic (LP), variant of unknown/uncertain significance (VUS), likely benign (LB), or benign (B).^2^ In the context of these categorizations, “likely” corresponds to a >90% confidence that an alteration is either pathogenic or benign.

P/LP designations denote those variants associated with human disease which are well understood and may be clinically actionable, either via (1) an FDA-approved therapy for the patient’s tumor type, (2) an off-label therapy for the specific biomarker, or (3) an investigational agent in a controlled clinical trial. P/LP variants can influence clinical decision-making for individual or familial screening, preventative surgery, treatment response monitoring, and treatment recommendations, and just as importantly, to avoid any unnecessary intervention. VUS is a classification of exclusion, where those alterations that either lack sufficient key pieces of scientific evidence or present conflicting evidence regarding their functional characterization or clinical significance are included in this grouping. A VUS has an undetermined association with a disease and an unknown impact on treatment and patient outcomes. Benign variants known to not contribute to disease development are often detected in healthy individuals and within various frameworks, are typically filtered entirely from final diagnostic outputs to mitigate overwhelming amounts of reported information. In the clinic, it is recommended that only P/LP variant classes be used in patient management, thereby creating a practical actionability threshold or binary cut point for intervention between the LP and VUS classifications.^3^

Soon after in 2017, a joint consensus between AMP, the American Society of Clinical Oncology (ASCO), and the College of American Pathologists (CAP) released guidelines that proposed a 4-tiered system to categorize alterations based on clinical significance. Within this framework, tier I (T1) variants were defined as those with strong clinical significance, tier II (T2) as variants with potential clinical significance, tier III (T3) as variants of unknown clinical significance, and tier IV (T4) as benign or likely benign variants.^4^

Recognizing the lack of agreement between recommendations, a joint consensus between the Clinical Genome Resource (ClinGen), the Cancer Genomics Consortium (CGC), and the Variant Interpretation for Cancer Consortium (VICC) released its own guidelines in 2022, which addressed the classification of pathogenicity of somatic variants in cancer (also referred to as oncogenicity).^5^ This group noted that prior guidelines lacked unambiguous and comprehensive standards to classify the oncogenicity of somatic alterations.

Orthogonal to sensitivity and specificity, the consequence of variant annotation itself has direct impact on patient care. Appropriate interpretation of NGS data requires specialized knowledge and expertise in molecular genetics, bioinformatics, and clinical medicine, where the lack of standardization in variant annotation can lead to significant discrepancies in clinical practice and patient outcomes. That said, there currently is no singular framework in the industry for standardized variant reporting and classification.

## Context of pathogenicity classification

It is critically important to appreciate the proposed scope of a platform before a result itself should be scrutinized. Testing intended to impact clinical decision-making must be extensively validated for making diagnostic, prognostic, or therapeutic judgements, while assays designed for investigational discovery or translational research are not as rigorously regulated; therefore, the same apparent result from each domain must be interpreted with the appropriate perspective. Irrespective of the assay size or underlying chemistry, the fundamental process of inferring the biological gene product by measuring the genome is rather imprecise for the bulk of variants; thus, pathogenicity prediction is largely inference.

The basic understanding that genomics is an interdisciplinary field offers a significant perspective on the classification of variant pathogenicity. The intended use or niche application of a diagnostic test determines how identified alterations are used in clinical practice. Assays can be broadly partitioned into those which detect either *non-cancer-related variants* (eg, *CFTR* p.F508del is universally accepted as pathogenic, but solely as a mutation that results in cystic fibrosis and has no known association with oncology) or *cancer-related variants*. The latter needs to be further segregated into testing intended to profile patients without cancer (for which familial predisposition, allelic penetrance, and probanding are relevant in determining hereditary cancer risk) vs. patients with cancer (for which cancer drivers and resistance mutations are relevant for treatment decision insights). Relative to the medical context, perhaps “pathogenic” could be interpreted more simply as “relevant.” Associations with elevated cancer risk are not relevant in patients already with late-stage cancer diagnoses. Pathogenic variants in the setting of early cancer detection may be construed as irrelevant in the treatment selection stage, which should be approached carefully in the context of monitoring treatment response via minimal residual disease (MRD). In fact, reporting of incidental pathogenic findings on somatic cancer tests is often not consented to by patients. Variant pathogenicity classification is essential in each these contexts and reported genomic information should be curated specifically to the intended use for patient care.

Further, the NGS diagnostic arena is currently saturated with tissue biopsies and liquid biopsies, both with and without the requirement of a matched-normal patient specimen, and all with varying analytic performance metrics. These platform dissimilarities add to the challenge of annotating the many variant classes which could be reported: single-nucleotide substitutions, short insertions and deletions (indels), copy number aberrations (gains and losses), and structural rearrangements. Bioinformatic methodologies could reasonably annotate a single exon deletion event as either a large indel (*BRCA1* c.5053_5136del), a copy number loss (*BRCA1* exon 18 loss), or a small structural rearrangement (*BRCA1* [exon 1-17] - *BRCA1* [exon 19-22]), each following independent logical annotation paths. These computational methods can vary between assay types and providers, conflating both the aggregation of big data and the interpretation of an individual patient result.

## Data used for pathogenicity classification

**Figure 1.**
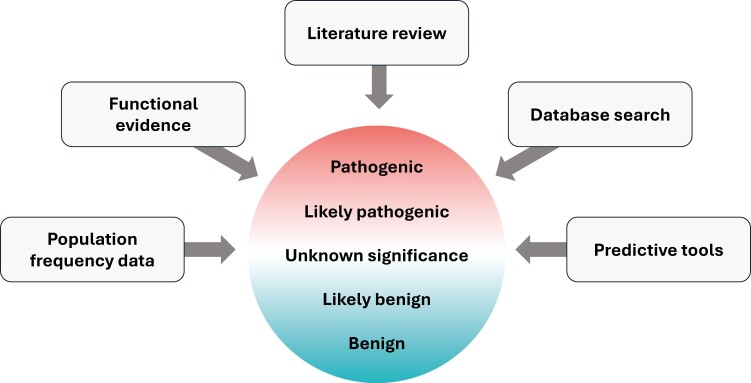
All available evidence should be included for the most accurate variant pathogenicity determination.

Pathogenicity interpretation based on the most current and comprehensive understanding of a variant is a cornerstone of a validated diagnostic. Classically—and especially for germline alterations—functional characterization has largely been inferred from an individual’s family history, which remains one of the most valuable sources of clinical information, though these data are generally not readily available in aggregate. In principle, though, all accessible data regarding pathogenicity should be evaluated for each variant, including any available literature, population frequency data, functional studies, clinical data (including n-of-one case reports), inferred protein impact, and predictive tools (Figure 1). However, not all data should be weighted equally, though the optimal framework to combine or score evidence items is not yet clear and access to these evidence items is not equal between laboratories.

### Literature review

A thorough review of the peer-reviewed scientific and medical literature should be performed when a diagnostic provider determines pathogenicity. This exercise should assess whether a variant has been functionally characterized, reported in the context of cancer, or has been associated with increased cancer predisposition. Functional studies can include both clinical and preclinical evidence, but preclinical results must be used cautiously. It is important to acknowledge that studies have appropriate controls and that results are compelling. Annotations including the position, gene symbol, transcript identifier, and reference genome assembly should match in order to ensure continuity. When literature evidence is aggregated, overlapping authorship or institutions should not be neglected, as this could lead to duplicate counting of data. Further, n-of-1 case reports should be examined with caution, but not excluded entirely—data from these sources are more applicable to novel variants or emerging resistance mechanisms than to common drivers. Routine literature review is essential, as evidence for or against pathogenicity will generally become available via the literature before those data are incorporated into public databases.

### Databases and real-world outcomes

One of the first strategies taken to identify determine pathogenicity is to search for available annotations in large population datasets. The Genome Aggregation Database (gnomAD), 1000 Genomes Project, the National Institutes of Health’s dbSNP, and other similar exemplars which aggregate population data from multiple sources provide information about alternative allele frequencies at many loci in very large cohort.^6-8^ These population databases are frequently referenced by bioinformatic pipelines to filter out common benign variants. However, though inter-database concordance is generally high for well-supported variants, annotation variability for rare variants can lead to ambiguity. These comprehensive global cohorts also underrepresent ethnicity-specific alleles. It should be noted that errors in classification can be introduced by the inclusion of canonical cancer-associated somatic alterations in these datasets and that these cohorts can erroneously include alterations which arise from clonal hematopoiesis in otherwise healthy individuals. Additional annotation steps like inter-database comparisons are typically advised to mitigate flawed annotations.

The Catalog of Somatic Mutations in Cancer (COSMIC), Cancer Hotspots, and My Cancer Genome are instances of commonly used cancer-specific data repositories that provide prevalence information about variants across a range of pathological diagnoses.^9-11^ Despite their frequent usage, entries are not all curated or reviewed, leading to potentially suboptimal quality of genomic data.

Contributive data sources such as ClinVar and Clinical Interpretation of Variants in Cancer (CIViC) are crowdsourced knowledgebases that make assertions about pathogenicity and therapeutic relevance of both germline and somatic variants.^12,13^ These assertions aim to summarize collections of evidence for a given alteration in the context of a specific cancer type. In all cases, the levels of evidence and submitting bodies should be evaluated for quality.

Private institutional, or in-house, databases are typically proprietary but have the potential to be the most descriptive. Hundreds of thousands of specimens tested on a single platform and held to the same quality control specifications while retaining a consistent annotation schema can power statistical discovery and serve as a valuable training set for computational tools. Clinical laboratories are generally encouraged to contribute their curated findings to open-access databases to continue annotation standardization, but patient consent, intellectual property, and other technical aspects need to be considered.

Philosophically, patient outcomes are the most important datapoints in clinical oncology. If used thoughtfully, survival metrics from real-world data (RWD) can not only validate established biomarkers but also inform the development of new ones.^14,15^ Routine clinical RWD rare arguably the most useful large-scale functional studies, especially for the mass amount of incidental VUS findings. If a particular variant is strongly associated with severe disease or poor treatment response, this can provide sufficient evidence for classifying the variant as pathogenic event without precise mechanistic evaluation. Variants that affect drug metabolism or treatment efficacy may be classified as pathogenic if they reliably predict adverse pharmacological response or treatment failure.

### Computational predictive algorithms

Many in silico algorithms are publicly or commercially available to aid in the classification of variant pathogenicity, assessing impact at the nucleotide, amino acid, and protein levels.^16^ Performance of computational predictive algorithms varies widely, where tools like AlphaFold aim to predict missense effects on protein structure^17^ and others like SpliceAI attempt to predict splicing variations.^18^ Interpreting these predictions, though, can be challenging.

In-house machine learning (ML) models trained on millions of alterations detected on a single platform and hundreds of well-annotated features including disease, co-occurring variants, somatic status, mutational signatures, and genome-wide scars may prove to be more predictive than tools that utilize heterogenous public data. Despite the potential, and even though evaluation of algorithms against pathogenicity classifications shows positive correlation and fair predictive values, it is currently recommended that computational predictive algorithms be considered in conjunction with other evidence types and not as independent support.^19^

## Axes of pathogenicity interpretation

**Figure 2.**
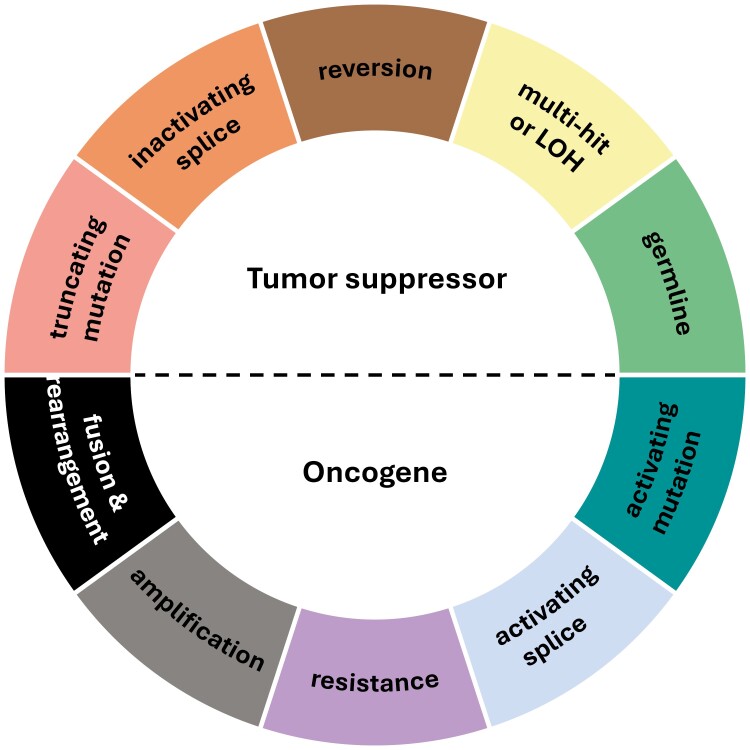
Pathogenic functional status is inferred from many data points and should be examined across multiple axes.

The term “pathogenicity” is highly dimensional, meaning that there are multiple axes upon which its definition should be interpreted. Within each domain, erroneous assignment of alteration pathogenicity and between-laboratory variability should be mitigated, but medical oncologists, genetic counselors, and bioinformatics scientists each infer pathogenicity by examining different criteria. As more NGS diagnostics become available and, more importantly, as their fundamental capabilities diverge, pathogenicity classification should include as many relevant data points as a platform has the ability to allow. Rather than using a linear decision-tree, these, among many other axes, should be considered in context, to determine the classification (Figure 2).

### Tumor suppressor vs. oncogene

At the highest-level, genomic oncology largely stratifies genes into either oncogene or tumor suppressor functions. This “class” model implies that the groupings have opposite roles—where loss-of-function (LOF) mutations in tumor suppressors and gain-of-function (GOF) mutations in oncogenes are canonically considered pathogenic—and dysregulation of wild-type function in either can lead to cancer development. Plenty of instances exist, though, where the mutation type directly contradicts the expected protein behavior. For example, frameshifts in the C-terminal of *PIK3CA*, a well-established oncogene, are novel activating oncogenic events that sensitize tumors to anti-PI3KA treatment.^20^

Further, genes do not always fit nicely into either category and depending on the disease and tissue type, a single gene may encode a protein that functions as either tumor suppressor or oncogene. Mutations that are characteristically annotated as pathogenic do not depend only on the gene, but the combination of gene and its context-dependent role in the specific disease.^21^ Classically, the highly conserved NOTCH signaling pathway, namely with respect to the *NOTCH* receptors (*NOTCH1-4*), is implicated in both biologic functions depending on cell type, developmental stage, or target genes. Germline mutations in the NOTCH pathway are associated with a variety of non-cancer related hereditary syndromes, but both activating mutations in the negative regulatory region or PEST domain and inactivating mutations in epidermal growth factor-like repeats of the extracellular domains should be interpreted as pathogenic within the context of cancer. The tumor-suppressive role of *NOTCH* has been demonstrated in both solid tumors and myeloid malignancies. Similarly, oncogenic functions have been described in both solid tumor and myeloid diseases. Perhaps even more enigmatic, exemplified in gliomas, is evidence that *NOTCH* can function as both tumor suppressor and oncogene even within the same tissue type,^22,23^ which further blurs the delineation between these gene classes.

### Effects on splicing

Predicting the functional significance of LOF splice events in tumor suppressors and GOF splice events in oncogenes can be challenging, impacting potential clinical significance. The distance from a splice junction and the alignment justification (3ʹ or 5ʹ) have significant impacts on pathogenicity interpretations of splice events and can even dictate whether they are reported at all. For example, *APC* c.835-8A>G, which is 8 nucleotides 5ʹ of the splice acceptor site and outside the typical reporting window of 2-3 bp, weakens the native splice acceptor site and may result in the creation or strengthening of a novel splice acceptor site.^24^

Generally, alterations that impact splice recognition and splice donor sites of tumor suppressor genes are considered LOF and are annotated as pathogenic. However, GOF splice alterations that result in constitutive signaling are even more likely to be oncogenic drivers. *MET* exon 14 skipping (METΔ14)—where an *MET* isoform lacks the intracellular juxtamembrane domain—is the result of any of hundreds of unique splice events and portends therapeutic response to anti-MET targeted therapy.^25^ In glioma, *EGFR* splice variants including deletion of *EGFR* exons 2-7 (EGFRvIII) result in ligand-independent constitutive signaling to downstream pathways.^26^ AR-V7, a splice variant of *AR* where a cryptic exon 3 leads to truncation of the ligand binding domain, demonstrates lack of response to hormonal therapy but causes no impact on response to taxane-based treatments in prostate cancers.^27^ Thoughtful consideration by the diagnostic provider of canonical, novel, and cryptic splice isoforms needs to be given to both tumor suppressors and oncogenes to report the most clinically meaningful functional effect.

### Mutation interactions and resistance

Knudson’s two-hit hypothesis posits that both alleles of a tumor suppressor must be inactivated to result in a phenotypic change.^28^ This implies that a diagnostic test needs to be able to determine whether an alteration is homozygous or heterozygous in order to be annotated as pathogenic, but this categorization is not interpreted easily from raw NGS data. An LOF sequence alteration detected on either the maternal or paternal allele should be interpreted differently from that same alteration detected with homozygous status.

Similarly to zygosity determination, but distinctly different, is the capability of an assay to determine whether multiple alterations are in cis (same allele) or trans (different alleles). There is ample evidence of an LOF alteration occurring on one allele and a separate LOF alteration occurring on the other; the overall pathogenicity impact in this case is greater than the sum of the parts. Likewise, an LOF alteration detected on a single allele combined with a loss of the other allele (loss of heterozygosity [LOH]), should be interpreted as homozygous. This is especially pertinent for synthetic lethality treatment strategies like those targeting PARP inhibition for bialleleic *BRCA1/2* loss^29^ or PRMT5/MAT2 inhibition for bialleleic *MTAP* loss.^30^

**Figure 3.**
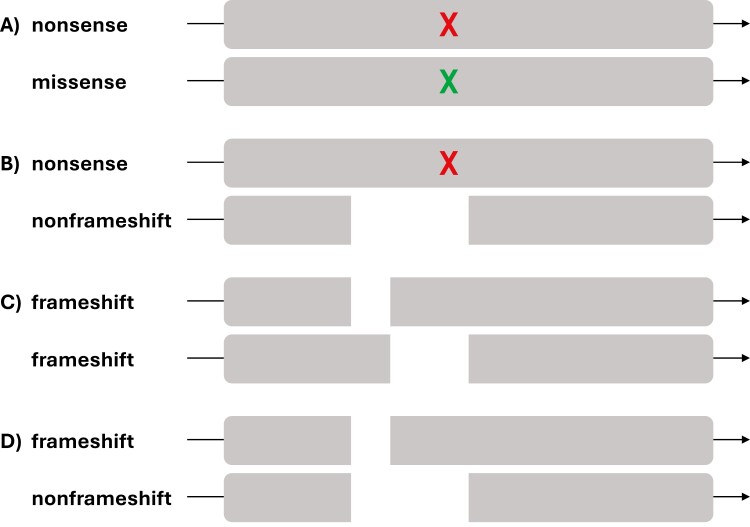
Mutations that revert a DNA sequence back in-frame can be among the following: (A) sites of truncating nonsense mutations where additional base substitutions result in non-truncating changes, (B) sites of truncating nonsense mutations where additional encompassing nonframeshift deletions result in non-truncating changes, (C) sites of truncating frameshift mutations where additional nearby frameshift mutations result in non-truncating changes, or (D) sites of truncating frameshift mutations where encompassing nonframeshift deletions result in non-truncating changes.

Perhaps the most complicated alteration type to detect and effectively report are reversion mutations. As seen in the *BRCA1/2* genes when exposed to PARP inhibition, reversion mutations are secondary mutations on a mutant allele that revert the initial frameshift mutation back in-frame to produce a functional protein product.^31^ By definition, reversions are always in cis, but this is not regularly apparent from raw NGS data. Corrective frameshifts that revert a neighboring frameshift (pathogenic reverting pathogenic) should be seen in cis, but replacement nonframeshift deletions that revert an encompassed nonsense or frameshift alteration (non-pathogenic reverting pathogenic) would be seen in trans. This type of acquired resistance has profound impacts on therapy selection, despite its biological function as anti-pathogenic (Figure 3).

Acquired resistance mutations in oncogenes looks quite different from reversions. *EGFR* T790M—the exemplary “gatekeeper” resistance mechanism to first-generation EGFR tyrosine kinase inhibitors—should always be seen in cis with another EGFR driver mutation like L858R or exon 19 nonframeshift deletion.^32^ Resistance to anti-ALK targeted therapies in NSCLC manifests as missense mutations (most frequently as L1196M and G1269A), but always in cis with sequence data indicating an ALK fusion event.^33^ The capability to determine cis/trans status partly relies on sophisticated bioinformatic methods and partly on assay design. Read length is typically the gaiting factor, but alterations located nearby (within 10-20 bps) should be annotated accordingly.

### Copy number thresholds

In principle, aneuploidy presents as gains and losses of combinations of parental alleles. These alterations can contribute to cancer development and progression by affecting the expression of key genes, but the relationship between copy number and expression is nonlinear and, in most cases, unknown. Reporting on the total landscape of genomic aneuploidy for each patient would be overwhelming and often clinically meaningless; therefore, providers utilize thresholds to report losses in tumor suppressor and amplifications in oncogenes. These thresholds differ between platforms and method, which leads to confusion translating to clinical significance.

Heterozygous copy number losses of tumor suppressors are often considered relevant in the diagnostic setting of hereditary cancer syndromes, whereas only homozygous losses are annotated as pathogenic by diagnostics in the treatment selection stage. Therefore, a heterozygous loss may be absent from the final output of many clinical reports.

Oncogene amplification is stratified on two distinct axes: focality and magnitude. Focality refers to the size of the amplicon, where focal amplifications of some oncogenes are widely appreciated as distinct therapeutic opportunities from polysomy or arm-level gains. Magnitude, or the degree of amplification, is further complicated by interactions between alterations. The capability to distinguish amplification of a mutated oncogene from amplification of the wild type is not standardized. Further, the pathogenicity of oncogene gains outside of *HER2* remains rather unclear—until recently, anti-HER2 treatment was correlated with IHC scores of 3+, but new data suggest HER2-low (correlation with IHC scores of 1+ or 2+) is also clinically actionable.^34^ Questions remain surrounding other oncogenes including *MET* and their amplification thresholds for druggability.

### Rearrangements and chimeric fusions

Large-scale chromosomal rearrangements are an entire class of alteration, typically detected using dedicated methodologies. Annotations applied to these events can differ drastically between platforms. Any functional truncations of tumor suppressors are commonly annotated as pathogenic. Internal deletion and some internal duplication events in these genes should be considered as pathogenic, whereas 5ʹ or 3ʹ duplication events are generally not. Kinase domain duplications of oncogenes should be annotated as pathogenic, especially when functional data supports the claim.

The word “fusion” is often used interchangeably with “rearrangement” for erroneous simplification of complex results. A fusion is a particular result of a rearrangement event which should predict a viable chimeric transcript, and, therefore, a potential novel chimeric protein. Specific rearrangements that result in *BCR*-*ABL*, *ELM4*-*ALK*, and *KIF5B-RET* are examples of well-characterized hybrids of kinase genes that are known to drive tumorigenesis.^35^ More recently, non-specific fusion pairs where *NTRK* (*NTRK1-3*) are the 3ʹ partner have demonstrated response to kinase inhibitors.^36^

Fusions should meet criteria specified by the assay, including strand, frame, orientation, and relevant functional domains. On DNA, fusions generally require raw read orientation of the two genes to be in strand, the conjoined exons to be in frame, and active domain to be positioned on the 3ʹ end. However, these strand and frame considerations may be absent for RNA-based platforms. Specific curation of gene fusion partners and breakpoints within common domains (eg, *ALK* intron 19) should help refine pathogenicity.

### Germline status

When annotating alterations, it is becoming increasingly important that a diagnostic be able to differentiate between germline and somatic, and perhaps even more important to segregate tumor somatic from those arising from clonal hematopoiesis. Identifying clinically relevant germline mutations, particularly within tumor suppressors (as there are exceptionally few pathogenic hereditary mutations in oncogenes, with *RET* and *EGFR* T790M as rare examples), is important not only for the patient but also for blood-relatives, who may be at increased risk of disease development. Routine use of large NGS panels not only discovers common germline variants but also enables the identification of rare germline susceptibility alterations. These mutations occur at a low frequency in the general population and are, therefore, more difficult to definitively classify, but can also be associated with an increased risk of developing a disease.

Diagnostic providers utilize different germline filtering methodologies. Some platforms sequence tumor alongside patient-matched normal specimens, but these normals can be contaminated with non-trivial levels of tumor DNA. Computational filtering methods are generally based on common population data and make the assumption that alterations annotated as benign are benign for the entire population. Ancestry and haplotype dependencies, which are important for precise germline determination, are generally excluded from naïve methods. Variant allele frequencies (VAF) near 50% are often considered to be germline, but this often-overlooked assumption is predicated on a diploid genome with no aneuploidy. In fact, the ESMO Precision Medicine Working Group recommends that variants with VAF > 20% (substitutions) and > 30% (indels) be considered for confirmatory germline testing.^37^ Similarly, though somatic mutations are typically detected at lower VAF, factors including tumor content (the relative amount of tumor versus normal DNA interrogated in a given specimen) can shift somatic VAF around 50% or even higher. More sophisticated computational germline filters which increase accuracy are multiparametric, incorporating VAF, tumor content, aneuploidy, and allelic imbalance.^38^

It is worth noting that clonal hematopoiesis alterations, which occur in blood progenitor cells and offer that cell linage a fitness advantage, are somatic, but not in the tumor genome and, therefore, not relevant to the progression of a solid tumor. The potential for interpretation interference with tumor somatic mutation calls is high, particularly in exemplars like *JAK2* V617F or very low VAF alterations in *DNMT3A*, *TET2*, *ASXL1*, *TP53*, and *SF3B1*. This obfuscates analyses of tumor dynamics as a predictor of disease progression or treatment response. However, such mutations are still clinically meaningful in that they have been linked to emergent hematological disorders and elevated cardiovascular disease risk and should prompt appropriate patient referrals.^39^ Validated workflows or computational methods to discern the origin of reported alterations is vital.

### Genome-wide signatures

Diagnostic oncology has evolved from detecting simple biomarkers to more complex and holistic genome-wide signatures, which adds another layer of complexity regarding pathogenic annotations. Implementation of binary thresholds onto continuous signatures is too complicated to address sufficiently in this review; however, a few considerations are worth highlighting.

Accurate determination of microsatellite instability (MSI), tumor mutational burden (TMB), and homologous recombination deficiency (HRD) are partially dependent on correct pathogenicity classifications of all alterations within a specimen.^40-42^ Efforts exemplified by the Friends of Cancer Research TMB Harmonization Project aimed to provide guidelines for how bioinformatic filters, including germline annotations, should be applied to computational TMB methodologies.^43^ Algorithms to detect MSI and HRD incorporate pathogenic variant status with other datapoints including aneuploidy, LOH, and zygosity.^42,44^

## Conclusion

Variant pathogenicity assignment is not a straightforward process—many groups have offered broad guidance, but there is no current widely accepted mandate to sufficiently standardize the process. For now, generalizable rules for pathogenicity are scalable and are largely followed, but as personalized oncology becomes more precise, these rules will need to be further curated, standardized by cancer genome experts, and universally accepted. Some groups have already implemented variant curation expert panels (VCEP) to accelerate the interpretation of pathogenicity.^45^ The most accurate assessments will take into account all of the axes upon which the term “pathogenicity” could be interpreted (Panel 1).

Panel 1.  **Strategies for determining pathogenicity**

I. Medical context
II. Available data
  A Literature review
  B Databases and real-world outcomes
  C Computational predictive algorithms
III. Axes of “pathogenicity”
  A Tumor suppressor vs oncogene
  B Effects on splicing
  C Mutation interactions & resistance
  D Copy number thresholds
  E Rearrangements and chimeric fusions
  F Germline status
  G Genome-wide signatures

The practice of accurately classifying—and even reclassifying—variant pathogenicity has obvious advantages for patients and healthcare providers. It can help to refine understanding of the genomic basis of disease, but more importantly, accurate classification can better inform clinical management decisions. This knowledge can inform the development of new treatments and prevention strategies and better identify which patients would benefit. Finally, reclassifying alterations can reduce uncertainty and anxieties surrounding VUS for patients and their families. Reclassifying a VUS as P/LP or B/LB can provide a more definitive answer and help to alleviate some of this uncertainty.

Importantly, the classification of alterations is not etched in stone. Prior to 2005, *EGFR* T790M was simply just another VUS, but is now universally recognized as a resistance mechanism to first generation anti-EGFR targeted therapies. As the dynamic research base evolves and more diagnostic testing is performed, new data regularly emerge, which can lead to the reclassification of an alteration anywhere on the spectrum of pathogenicity. This should be an ongoing practice performed by all diagnostic providers and the oncology community as a whole should expect molecular diagnostic results to change appropriately as scientific knowledge progresses.

When there is a strong biological association between alteration and pathology, it is possible to accurately classify an alteration after only a few observations, but this straight-forward approach is generally not afforded for uncommon or family-specific alterations. Although they are typically individually rare, the vast majority of patients do harbor one or more VUS and the increasing use of multigene NGS panels has led to a rapid increase in the probability of identifying VUS. Insufficient functional or clinical studies or the overall lack of available data create an inability to apply meaningful statistical methods on a large number of these rare alterations. The challenge of VUS interpretation is particularly relevant for genes that have a high degree of functional complexity and can be made more nuanced by the interplay between genomic and epigenomic factors. The current standards and guidelines are deliberately broad to allow for alteration classifications outside the scope of oncology, but this ambiguity ultimately can lead to inaccurate or conflicting status assignments in variant interpretation, muddying the already murky waters for care providers.

The likelihood of reclassification of VUS can vary depending on several factors, including the specific gene, the type of alteration, and the available evidence. In the literature, exceptionally few alterations initially classified as benign or pathogenic get reclassified. This indicates that with a definitive classification (P or B) it remains unlikely that reassessment will have an impact. The vast majority of reclassifications constitute VUS which are demoted to B/LB which remain under the actionability threshold and, therefore, do not impact patient care. Though much rarer, some still are VUS which are reassigned to P/LP or are P/LP demoted to VUS,^46,47^ which cause confusion to the treating medical teams, patients, and even payers. As of today, the rate at which new VUS are discovered still outpaces the rate at which known VUS are reclassified.

In order to mitigate the bias of any prior misclassification, with every reanalysis, even for P/LP or B/LB alterations, any given alteration should be assumed to be classified as a VUS until sufficient data emerges to alter that designation. Historically, reinterpretation has followed a reactive model, which lends itself to reporting of potentially outdated classifications. Importantly, when alteration reclassification causes a jump between sides of the LP/VUS boundary, there could be significant implications. A regular, more proactive framework for alteration reclassification may prove to be more valuable, though this would be a significant resource constraint. Knowledge gaps in alteration reclassification present significant limitations for genetic counselors and clinicians in the clinical management of patients. Along with improved education, it is imperative both for care teams to stay informed on the latest research and guidelines regarding pathogenicity interpretation and for diagnostic providers to proactively amend patient results when revised pathogenicity assignments are clinically meaningful.

## Data Availability

No new data were generated or analyzed in support of this research.
